# Inhibition of PI3K Isoform p110γ Increases Both Anti-Tumor and Immunosuppressive Responses to Aggressive Murine Head and Neck Squamous Cell Carcinoma with Low Immunogenicity

**DOI:** 10.3390/cancers13050953

**Published:** 2021-02-25

**Authors:** Kelvin Anderson, Nathan Ryan, Anastasia Alkhimovitch, Arham Siddiqui, Steve Oghumu

**Affiliations:** 1 Department of Pathology, The Ohio State University Wexner Medical Center, Columbus, OH 43210, USA; anderson.2325@osu.edu (K.A.); ryan.1046@osu.edu (N.R.); alkhimovitch.1@buckeyemail.osu.edu (A.A.); siddiqui.120@osu.edu (A.S.); 2 Division of Anatomy, The Ohio State University Wexner Medical Center, Columbus, OH 43210, USA

**Keywords:** PI3K, HNSCC, immune checkpoint, p110γ, PD-L1

## Abstract

**Simple Summary:**

Poorly immunogenic head and neck squamous carcinomas (HNSCC) remain difficult to treat due to poor response rates to immunotherapy. Inhibition of the PI3K catalytic subunit p110γ, which is expressed in leukocytes and some HNSCCs, has shown promise in treating HNSCC; with clinical trials underway to gauge its effectiveness. However, the effect of PI3K p110γ inhibition on the host immune system in poorly immunogenic HNSCC has not been fully described. In this study, our group characterized the immune response to poorly immunogenic HNSCC in the absence of PI3K p110γ using an orthotopic mouse model with the MOC2 cell line. We found that mice lacking p110γ did not demonstrate significantly different tumor growth or metastasis, though we observed substantial elevation in both anti-tumor and immunosuppressive activity at the primary tumor site. Our results indicate that PI3K p110γ inhibition may potentially enhance anti-tumor immunity against poorly immunogenic HNSCC if administered with checkpoint inhibitors.

**Abstract:**

HNSCC is the sixth most common cancer, with around 650,000 new cases yearly. Gain of function mutations in the PI3K pathway are common in HNSCC, and inhibition of the PI3K p110γ subunit has shown promise in HNSCC treatment. However, given that PI3K p110γ plays an important role in myeloid and lymphoid immune cell function, it is essential to understand how PI3K p110γ inhibition affects the anti-tumor immune response independent of tumor cells. To elucidate PI3K p110γ function in HNSCC, we employed an orthotopic mouse model using poorly immunogenic and aggressive cell line MOC2 on *Pik3cg^−/−^* mice. We observed that wild-type and *Pik3cg^−/−^* mice displayed similar rates of HNSCC tumor growth and metastasis after 20 days following tumor injection. T-cell infiltration and intrinsic T-cell responses to MOC2 oral tumors were comparable between wild-type and *Pik3cg^−/−^* mice. Interestingly, the immune response of tumor-bearing *Pik3cg^−/−^* mice was marked by increased anti-tumor cytotoxic molecules (IFN-γ, IL-17)) by T-cells and immune checkpoint marker (PD-L1, PD-1) expression by myeloid cells and T-cells compared to tumor-bearing wild-type mice. Taken together, our findings demonstrate that inhibition of PI3K p110γ modulates tumor-associated immune cells, which likely potentiates HNSCC treatment when used in combination with selective checkpoint inhibitors.

## 1. Introduction

HNSCC is the sixth most common form of cancer and the ninth deadliest, with around 650,000 new cases a year and 330,000 deaths attributed to the disease worldwide [1]. Current estimates demonstrate that HNSCC accounts for almost 4% of cancer cases in the United States [2]. Because the genetics of HNSCC are considerably varied across patients, therapies must be tailored to each individual in order to ensure the best medical outcomes [3].

Phosphatidylinositol-3-kinase (PI3K) has emerged as a potential target for treating patients with HNSCC. There are four classes of PI3K that are categorized based on their protein structure and roles in cell biology, of which Class I PI3Ks are most well defined [4]. Class I PI3Ks are heterodimeric proteins consisting of a regulatory subunit and a catalytic subunit, of which there are four isoforms—p110α, p110β, p110γ, and p110δ [4]. Class I PI3Ks catalyze the phosphorylation of inactive phosphatidylinositol 4,5-bisphosphate (PIP_2_) to the signal transducer phosphatidylinositol 3,4,5-trisphosphate (PIP_3_) after activation from extracellular signaling compounds such as epithelial growth factor (EGF) in epithelial cells and CD28 in T-cells [5]. Downstream, Akt activation induces cellular changes that ultimately lead to cell growth, activity, and survival [5,6]. PI3K signaling has been shown to be important in a number of inflammatory and autoimmune diseases. For example, PI3K p110γ inhibition has been shown to confer a therapeutic anti-inflammatory effect in preclinical and clinical studies of asthma and COPD [7]. In vitro cultures of human HNSCC cells as well as mouse xenograft models indicate that PI3K signaling is linked to cancer radiation resistance, and PI3K inhibition has shown promise in potentiating the cytotoxic and anti-mitotic effects of radiotherapy [8,9]. This is an area that is undergoing active preclinical and clinical investigation [10]. The role of PI3K in resistance to irradiation involves several mechanisms, including elevated DNA damage repair through activation of DNA-protein kinases (DNAPK), and induction of hypoxia-inducible factor (HIF-1) dependent gene transcription [11,12].

The mitogenic and anti-apoptotic properties of PI3K activity results in HNSCC cancer cells tumorigenicity [13]. A meta-analytical study showed that 66% of examined clinical HNSCC cases demonstrate an increase in pathway activity due to aberrant expression of key mediators of the PI3K-Akt-mTOR signaling pathway, including downregulation of the repressor PTEN and upregulation in the activity of PI3K catalytic subunits [14]. Notably, whole-exome sequencing in a cohort of HNSCC patients showed that 30.5% of HNSCC tumors contained mutations in the PI3K-Akt-mTOR pathway, mostly in *Pik3ca*, *Pik3cg*, and *Pten* [15]. These studies associated increased PI3K-Akt-mTOR activity with poor prognosis. Due to the frequency of genetic aberrations that increase activity in the PI3K-Akt-mTOR pathway, there is a rationale for directly targeting frequently mutated mediators of this pathway to control the progression of HNSCC, including Class I PI3K catalytic subunits [15]. Currently, there are many clinical trials employing PI3K inhibitors as monotherapies against solid tumors, including a phase II clinical trial utilizing the p110γ inhibitor IPI-549 on locally advanced HNSCC [5,16]. However, suboptimal patient response rates have been observed in studies employing PI3K inhibitors as monotherapies on tumors exhibiting gain-of-function in this pathway, necessitating further exploration of their activity and usage [17].

On the other hand, selective PI3K inhibitors, such as IPI-549, have seen success in clinical trials when combined with other therapeutic strategies [18]. Such studies indicate that inhibition of PI3K p110γ in combination with other therapies could enhance anti-tumor immune function. For example, a recent study in a murine melanoma model showed that adoptively transferred CD8+ T-cells treated with IPI-549 exhibit increased cytotoxicity [19]. Further, IPI-549, in conjunction with anti-PD1 therapy, led to a stronger anti-tumor effect than either therapy alone in mouse models of HNSCC, lung, and breast carcinoma [20]. These findings are particularly intriguing because they indicate that PI3K p110γ may serve as a target of host-directed therapies that improve the anti-tumor immune response independent of PI3K p110γ expression by tumor cells.

Despite these encouraging findings, the PI3K-Akt-mTOR pathway in T cells, mediated through p110γ, has been shown to be critical for their development and functional responses [21,22,23,24]. Because PI3K inhibitors do not discriminate between cancer cells and healthy cells, it stands to reason that the suboptimal response to PI3K inhibition may be due to a deleterious global effect on normal cell functioning. Therefore, targeting PI3K p110γ could lead to undesirable changes in a host’s immune phenotype. For example, it has been shown that in some metastatic breast cancer patients, the use of PI3K inhibitors correlates with depletion of anti-tumor CD8+ T cells and expansion of suppressor cells [25]. Further, dual inhibition of p110γ and p110δ has been shown to abrogate murine CD8^+^ activity ex-vivo [19], while in-vivo demonstrated limited effect on poorly immunogenic HNSCC in a mouse model, even in conjunction with PD-L1 blockade [26].

At this point, significant gaps remain in the understanding of PI3K-Akt-mTOR signaling within the host’s immune cells in the context of HNSCC. While PI3K p110γ inhibitors have shown promise as a host-directed approach to improving the immune response against HNSCC, the global effect of PI3K p110γ inhibition in host immune cells is not well understood. Additionally, a primary factor of resistance to therapeutics targeting the PD-1-PD-L1 axis is low tumor immunogenicity [27]. Previous experiments have not demonstrated whether host-directed PI3K p110γ inhibition confers improved cytotoxic function against poorly immunogenic HNSCC tumors. Filling such gaps is necessary to fine-tune the administration of PI3K p110γ inhibitors against HNSCC, and elucidate the mechanisms that lead to low response rates.

In view of these findings, our group sought to investigate the effect of PI3K p110γ deficiency on host immune responses to poorly immunogenic HNSCC tumors using an orthotopic murine model with *Pik3cg^+/+^* wild-type (WT) mice and *Pik3cg^−/−^* mice. To establish HNSCC, we utilized the syngeneic C57BL/6J murine oral cancer cell line (MOC2), which is known to establish tumors with low immunogenicity, high metastatic incidence, and poor responsiveness to checkpoint inhibition [28,29]. MOC2 cells were derived from a tumor in the floor of the mouth of a mouse experimentally induced with an oral carcinogen 7,12-dimethylbenz(a)anthracene (DMBA); and next-generation sequencing of MOC2 cells used in this study demonstrates striking similarities to the aberrant genetic signature of human HNSCC [30]. Further, our orthotopic model used mouse HNSCC cell lines in immunocompetent mice, which possesses distinct advantages for the elucidation of immunological responses to HNSCC both systemically and in the tumor microenvironment. Our results showed that mice deficient in *Pik3cg^−/−^* did not display a significant reduction in tumor growth or survival compared to WT mice. However, we discovered remarkable differences in the expression of cytotoxic molecules and immunosuppressive markers that could potentially be exploited in combatting poorly immunogenic HNSCC.

## 2. Results

### 2.1. Host PI3K p110γ Inhibition Does Not Significantly Affect Tumor Growth and Metastasis in Mice Injected with MOC2 Oral Cells

**Figure 1:**
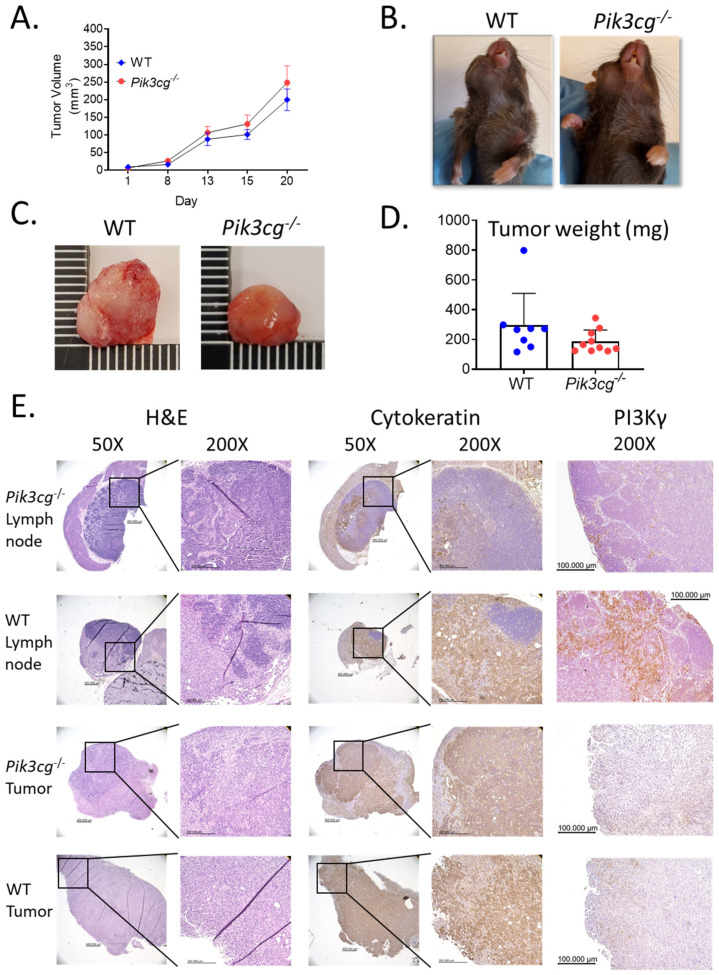
Host PI3K p110γ inhibition does not have a significant effect on tumor growth or metastasis in mice injected with MOC2 oral cells. (**A**) Tumor volume in WT and *Pik3cg*−/− mice over the duration of the experiment. (**B**) Images of WT and *Pik3cg*- mice. Tumors were located on the right buccal side of the mice. (**C**) Images of whole tumors excised from experimental mice at terminal sacrifice. (**D**) Tumor weights were taken from whole tumors removed at terminal sacrifice. (**E**) Representative hematoxylin and eosin and immunohistological images of lymph node and tumor sections from tumor-bearing WT and *Pik3cg*^−/−^ mice stained with AE1/3 pan-cytokeratin or PI3K p110γ antibodies and counterstained with hematoxylin at 50× and 200×. Lymph node sections demonstrate regions stained positive for pan-cytokeratin, indicative of regional metastasis.

To determine the involvement of host *Pik3cg* in the response against HNSCC, we injected an aggressive murine oral cancer cell line MOC2 into the right buccal mucosa of both WT and *Pik3cg^−/−^* C57BL/6 mice. As expected, based on previous studies utilizing this cell line, tumor growth occurred rapidly until mice reached early-removal criteria at 20 days. The rates of tumor growth in WT and *Pi3kcg^−/−^* mice assumed a similar trajectory. No difference was observed in tumor size based on caliper volume measurements (Figure 1A,B). This was not entirely surprising, given the rapid and aggressive growth pattern of this orthotopic HNSCC model. Our analysis of tumor weights demonstrated no statistically significant difference between groups, although the weights of tumors of *Pi3kcg^−/−^* mice appeared slightly lower than in WT mice (Figure 1C,D). In addition to their high growth rates, MOC2 cells are known to display high rates of lymph node metastasis, which is characteristic of aggressive human HNSCC. We, therefore, performed histological analysis of lymph nodes and tumors of tumor-bearing WT and *Pi3kcg^−/−^* mice to visualize tumor cell infiltration and metastatic tumor establishment. We observed similar tumor morphology and rates of lymph node metastasis in tumor-bearing WT and *Pi3kcg^−/−^* mice. (Figure 1E). PI3Kγ immunohistochemistry (IHC) staining showed mild positivity in tumors of WT and *Pi3kcg^−/−^* mice, while draining lymph nodes of tumor-bearing WT and *Pi3kcg^−/−^* mice showed moderate and very light positivity, respectively (Figure 1E). Taken together, our phenotypic and histological analyses of HNSCC tumors in WT and *Pi3kcg^−/−^* mice demonstrate that host PI3K p110γ inhibition did not have a significant effect on tumor growth and metastasis in the orthotopic MOC2 mouse model of poorly immunogenic HNSCC.

### 2.2. Tumor-Bearing Pik3cg^−/−^ Mice Do Not Possess Inherent Deficiencies in T Cell Activity

**Figure 2:**
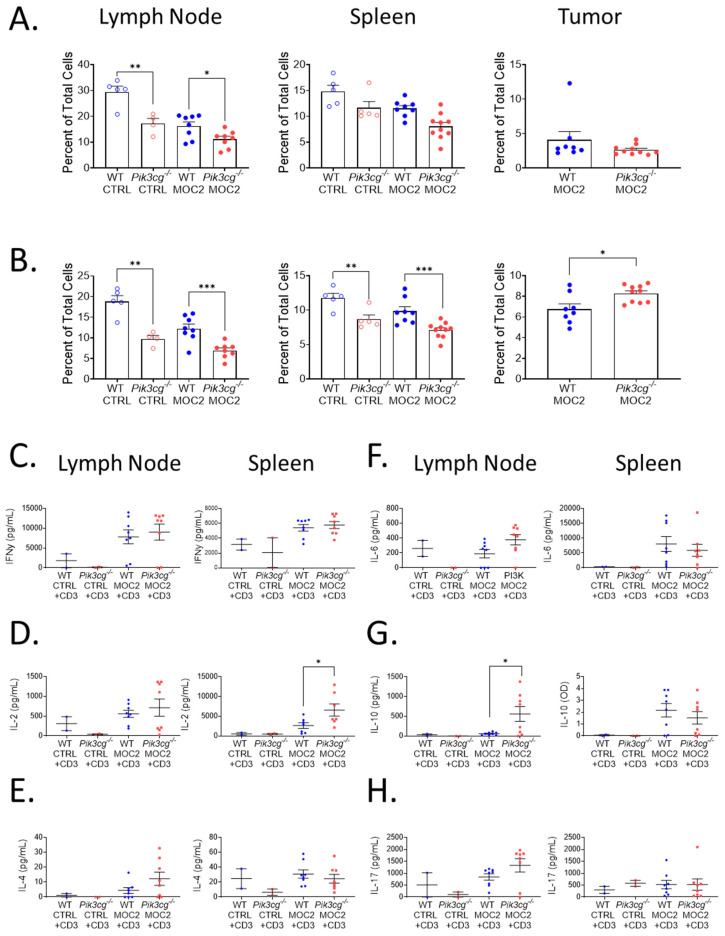
Tumor-bearing Pik3cg^−/−^ mice do not possess inherent deficiencies in T-cell activity. (**A**,**B**) Population frequency of (**A**) CD4^+^ T-cells and (**B**) CD8^+^ T-cells among total live cells within the lymph node, spleen, and tumor of WT and *Pik3cg^−/−^* mice as determined by flow cytometry. (**C**–**H**) Production of (**C**) IFN-γ, (**D**) IL-2, (**E**) IL-4, (**F**) IL-6, (**G**) IL-10, (**H**) IL-17 by CD3-stimulated T-cells isolated from the lymph node and spleens of experimental mice as determined by cytokine ELISA. * *p*-value < 0.05; ** *p*-value < 0.01; *** *p*-value < 0.005 between groups, as determined by Student’s *t*-test.

Because PI3K p110γ-mediated signaling has been described to be involved in T cell maturation and survival [23], we determined whether there were deficiencies in the anti-tumor immune function of T-cells of tumor bearing *Pik3cg^−/−^* mice. First, we analyzed CD4^+^ and CD8^+^ T cell populations in the lymph nodes, spleens, and tumors of tumor bearing and non-tumor bearing WT and *Pik3cg^−/−^* mice. As expected, in non-tumor bearing mice, proportions of CD4^+^ and CD8^+^ T-cells were significantly lower in lymph nodes and spleens of *Pik3cg^−/−^* mice compared to WT mice. This trend was also observed among T-cells in lymph nodes and spleens tumor bearing *Pik3cg^−/−^* mice compared to WT mice (Figure 2A,B). However, among CD8^+^ T-cells, we observed a marked increase in the infiltration of CD8^+^ T cells in *Pik3cg^−/−^* mice compared to WT mice. (Figure 2B).

To determine whether there were inherent deficiencies in T-cell ability to respond to oral tumor insult, we isolated T-cells from the lymph nodes and spleens of both tumor-bearing and non-tumor bearing mice and examined their ability to produce T-cells associated cytokines — IL-2, IL-4, IL-6, IL-10, IL-17, and IFN-γ —following CD3 stimulation. We found that IFN-γ, IL-4, IL-6, and IL-17 production by lymph nodal and splenic T-cells were comparable between both *Pik3cg^−/−^* and WT mice. However, we also observed an elevation of IL-2 by splenic T-cells and IL-10 by T-cells from the cervical lymph nodes of *Pik3cg^−/−^* mice, compared to T-cells from WT mice (Figure 2C–H). Taken together, we show that intrinsic T cell activity in the absence of host PI3K p110γ remains sufficient to engage in anti-tumor immunity. Further CD8^+^ T-cell infiltration into the HNSCC tumor microenvironment is enhanced in the absence of host PI3K p110γ.

### 2.3. Host PI3Kγ Inhibition Augments the Anti-Tumor CD8+ T-Cell Response to Poorly Immunogenic Experimental HNSCC

**Figure 3:**
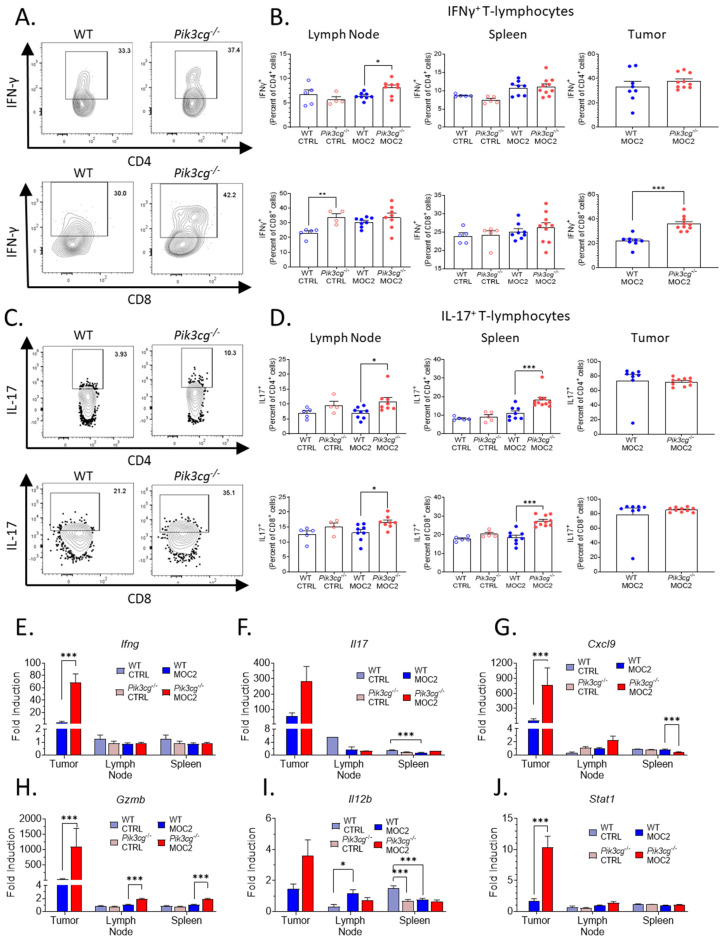
Host PI3Kγ inhibition augments the anti-tumor CD8+ T-cell response to poorly immunogenic experimental HNSCC. (**A**) Flow cytometry gating for CD4^+^ IFN-γ^+^ T-cells and CD8^+^ IFN-γ^+^ T-cells. (**B**) Population frequencies of CD4^+^ IFN-γ^+^ T-cells and CD8^+^ IFN-γ^+^ T-cells among parent T-cell populations in the lymph node, spleen, and tumor. (**C**) Flow cytometry gating for CD4^+^ IL-17^+^ T-cells and CD8^+^ IL-17^+^ T-cells. (**D**) Population frequencies of CD4^+^ IL-17^+^ T-cells and CD8^+^ IL-17^+^ T-cells among parent T-cell populations in the lymph node, spleen and tumor. (**E**–**J**) Expression of (**E**) *Ifng*, (**F**) *Il17,* (**G**) *Cxcl9*, (**H**) *Gzmb*, (**I**) *Il12b*, (**J**) *Stat1* within the sentinel lymph nodes, spleens, and tumors of experimental mice determined by RT-qPCR. * *p*-value < 0.05; ** *p*-value < 0.01; *** *p*-value < 0.005 between groups, as determined by Student’s *t*-test.

CD8^+^ T-cell tumor infiltration is associated with heightened anti-tumor immunity, and cytotoxic responses in HNSCC [31], and anti-tumor activity elicited by PI3K inhibition has been shown to be dependent on interferon-gamma (IFN-γ) and IL-17 [32]. Therefore, we investigated the effect of host PI3Kγ inhibition on intracellular production of IFN-γ and IL-17 by T-cells of experimental mice via flow cytometry. We observed a significant increase in intracellular IFN-γ expression by CD8^+^ T-cells in the tumors of *Pik3cg^−/−^* mice. No significant differences were observed in IFN-γ production by CD4^+^ and CD8^+^ T-cells in the spleens and lymph nodes of tumor-bearing mice (Figure 3A,B). We further found significantly enhanced *Ifng* gene expression in the tumors of *Pik3cg^−/−^* mice, though the expression was not different in the spleen or lymph nodes (Figure 3E). Because of the increased presence of IFN-γ^+^CD8^+^ T-cells and *Ifng* expression in the tumors of *Pik3cg^−/−^* mice, we more closely examined factors that could potentially underlie and contribute to the cytotoxic activity of this T-cell subpopulation. *Stat1*, which is involved in anti-tumor responses following IFN-γ stimulation, was much more highly expressed in the tumors of *Pik3cg^−/−^* mice compared to WT mice (Figure 3J). Additionally, *Gzmb,* an effector molecule involved in CD8^+^ killing of cancer cells, and *Cxcl9*, a chemokine important to CD8^+^ recruitment, were both highly elevated in the tumors of *Pik3cg^−/−^* mice compared to WT mice (Figure 3G, H). We also examined *Il12b*, which is known to promote IFN-γ production and cytotoxic activity by CD8^+^ T-cells [33]. Expression of this cytokine was not elevated significantly in any analyzed tissues, indicating that enhanced the IFN-γ production in *Pik3cg^−/−^* CD8^+^ T-cells was unlikely to be dependent on IL-12 signaling (Figure 3I). We also investigated intracellular IL-17 production by T-cells, which has been implicated in the recruitment and engagement of tumor-infiltrating lymphocytes [34]. We observed significant increases in IL-17 expression by both CD4^+^ and CD8^+^ T-cells in the spleens and lymph nodes of tumor-bearing *Pik3cg^−/−^*, though not in the tumor (Figure 3C,D). *Il17* expression was significantly elevated in the tumor, while its expression was low or undetectable for all mice in the lymph nodes and spleens (Figure 3F). Together, these findings indicate that host PI3K p110γ inhibition augments the anti-tumor CD8^+^ T-cell response to poorly immunogenic HNSCC.

### 2.4. PD-1 Expression by T-Cells Is Upregulated in Pik3cg^−/−^ Mice

**Figure 4:**
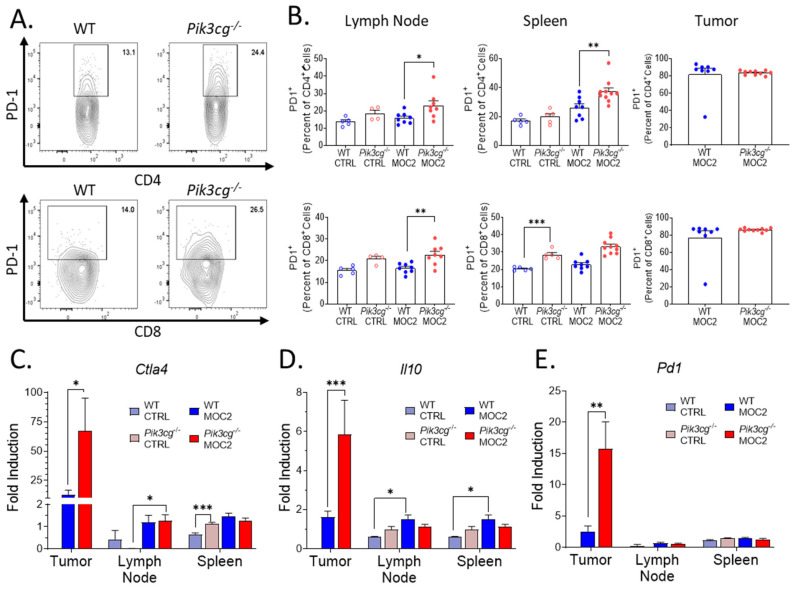
PD-1 expression by T-cells is upregulated in *Pik3cg^−/−^* mice (**A**) Flow cytometry gating strategy for CD4^+^ PD-1^+^ T-cells and CD8^+^ PD-1^+^ T-cells. (**B**) Population frequencies of CD4^+^ PD-1^+^ T-cells and CD8^+^ PD-1^+^ T-cells among parent T-cell populations in the lymph node, spleen and tumor. (**C**–**E**) Expression of (**C**) *Ctla4,* (**D**) *Il10,* (**E**) *Pd1*, and within the sentinel lymph nodes, spleens, and tumors of experimental mice determined by RT-qPCR. * *p*-value < 0.05; ** *p*-value < 0.01; *** *p*-value < 0.005 between groups, as determined by Student’s *t*-test.

Previous studies show that small molecule inhibition of PI3K p110γ enhances the efficacy of PD-1 blockade in HNSCC [20]. This led us to explore PD-1 expression by T-cells in tumor-bearing mice using our highly aggressive HNSCC model. We first examined the surface expression of PD-1 by CD4^+^ and CD8^+^ T-cell populations with flow cytometry (Figure 4A). We found that in tumor-bearing *Pik3cg^−/−^* mice, PD-1 expression was significantly higher in both CD4^+^ and CD8^+^ cells of the lymph nodes and spleens, though we did not observe this difference in the tumor microenvironment (Figure 4B). Our observations on PD-1 expression in *Pik3cg^−/−^* mice led us to further examine the potential for immune suppression in tumor-bearing *Pik3cg^−/−^* mice. Using RT-qPCR, we investigated the expression of lymphocytic immune checkpoint surface markers PD-1 and CTLA-4, as well as the immunosuppressive cytokine IL-10, via RT-qPCR in the lymph nodes, spleens, and tumors of experimental mice. While we saw no significant differences in the spleens and lymph nodes, *Ctla4, Il10,* and *Pd1* were significantly elevated in the tumors of *Pik3cg^−/−^* mice (Figure 4C-E). Taken together, these data demonstrate that T-cells of tumor-bearing mice display markers of tumor immune suppression in the absence of PI3K p110γ signaling.

### 2.5. PI3Kγ Inhibition Promotes Tumor Associated Myeloid Populations Which Express the Immunosuppressive Marker PD-L1 in the HNSCC Tumor Microenvironment

**Figure 5:**
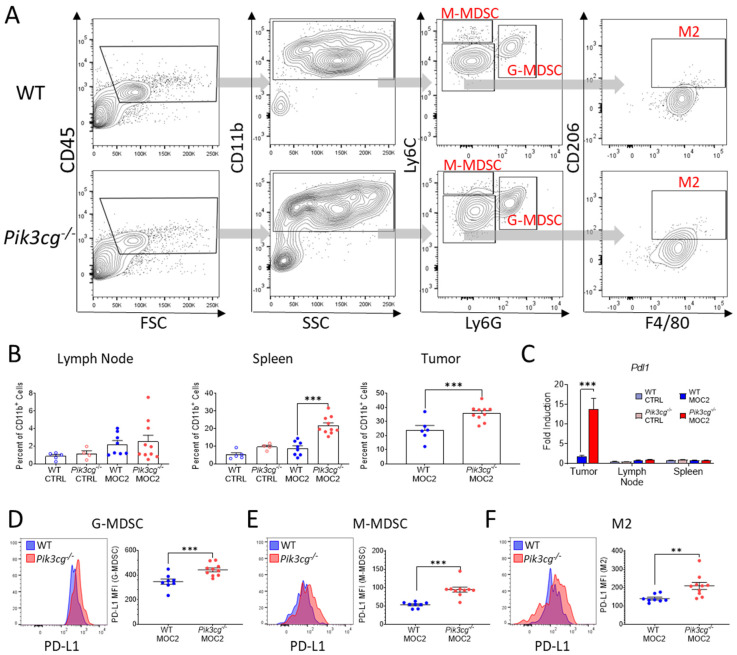
PI3Kγ inhibition promotes tumor-associated myeloid populations, which express the immunosuppressive marker PD-L1 in the HNSCC tumor microenvironment. (**A**) Flow cytometry gating strategy for Ly6C^+^ Ly6G^+^ granulocytic myeloid-derived suppressor cells (G-MDSCs), Ly6C^hi^ Ly6G^-^ monocytic MDSCs (M-MDSCs), and F4/80^+^ CD206^+^ Ly6G^-^ alternatively-activated macrophages (M2s) of experimental mice. (**B**) Population frequencies of G-MDSCs among CD11b^+^ populations in the lymph nodes, spleens, and tumors of experimental mice. (**C**) Expression of *Pdl1* within the sentinel lymph nodes, spleens, and tumors of experimental mice determined by RT-qPCR; (**D**–**F**) Mean fluorescent intensity of PD-L1 expression by (**D**) G-MDSC, (**E**) M-MDSC, (**F**) M2 populations in experimental mice accompanied by representative histograms demonstrating disparities in PD-L1 expression between WT and *Pik3cg^−/−^* mice.; ** *p*-value < 0.01; *** *p*-value < 0.005 between groups, as determined by Student’s *t*-test.

Immunosuppressive myeloid populations, including myeloid-derived suppressor cells (MDSCs) and alternatively activated macrophages (M2s), are known to be highly involved in aggressive and poorly immunogenic HNSCC, in part due to PD-L1 mediated suppression of anti-tumor lymphocytes [26,35]. Therefore, we analyzed Ly6C^+^ Ly6G^+^ granulocytic MDSCs (G-MDSCs), Ly6C^hi^ Ly6G^-^ monocytic MDSCs (M-MDSCs), and F4/80^+^ CD206^+^ Ly6G^-^ M2 macrophages, as well as their expression of PD-L1 (Figure 5A). Within the spleens and primary tumor sites of tumor-bearing *Pik3cg^−/−^* mice, there was increased infiltrate of G-MDSCs (Figure 5B), though no differences were observed in M-MDSC and M2 populations in any of the analyzed tissues (data not shown). We further found *Pdl1* gene expression to be significantly elevated in the tumors of *Pik3cg^−/−^* mice (Figure 5C). Notably, at the tumor site, all of the aforementioned populations showed significantly higher expression of PD-L1 in tumor-bearing *Pik3cg^−/−^* mice, suggestive of enhanced immunosuppression compared to WT mice (Figure 5D–F). In *Pik3cg^−/−^* mice, we also observed that M2 populations showed enhanced expression of PD-L1 in the spleens but not the lymph nodes, while G-MDSCs and M-MDSCs showed no difference in the spleens or lymph nodes (data not shown). Taken together, our data suggest a mixed tumor-associated immune response characterized by PD-L1 driven immune suppression by myeloid cells combined with pro- and anti-tumor CD8^+^ T-cell responses in the tumors of *Pik3cg^−/−^* mice.

## 3. Discussion

Poorly immunogenic HNSCC is associated with poor diagnosis and is extremely difficult to treat because of resistance to immunotherapies, including checkpoint inhibitors [36,37]. Therefore, novel approaches are required to overcome the challenges associated with the treatment of such frequent cases of HNSCC [38]. PI3K p110γ inhibition has presented itself as a novel means of combating HNSCC, although it has mostly been studied using pharmaceutical compounds that do not differentiate between healthy and tumor cell populations. Because we designed our experimental mouse study to only measure the effects of PI3K p110γ mediated signaling in host cells, we were able to focus on how PI3K p110γ inhibition affects the host immune response to HNSCC.

In this study, we have highlighted the effects of PI3K p110γ-mediated signal transduction on the immune response to aggressive HNSCC with low immunogenicity. By utilizing an orthotopic cancer model with the MOC2 cell line injected into WT and *Pik3cg^−/−^* mice, we have demonstrated that PI3K p110γ modulates host immune activity in a manner that likely potentiates the efficacy of anti-PD-L1 immunotherapy against HNSCC. While PI3K p110γ inhibition in host immune cells did not significantly lower tumor burden or influence lymph node metastasis, the immune response in the tumor microenvironment was substantially modulated in *Pik3cg^−/−^* mice. Remarkably, we observed increased expression of both anti-tumor cytotoxic and immunosuppressive markers in the tumor site of *Pik3cg^−/−^* mice, which may have resulted in counteracting effects on HNSCC development. Such findings may explain the suboptimal response rates of PI3K p110γ inhibition when employed alone against tumors in early phase clinical trials [17]. Although our study used a murine HNSCC model, we believe these results provide essential information that can potentially be applied to HNSCC patients in future studies.

Despite a lack of difference in tumor growth and metastasis, it was surprising to observe increased CD8^+^ T-cell tumor infiltration in tumor-bearing *Pik3cg^−/−^* mice, a phenotype generally associated with improved prognosis in human HNSCC [39]. These cells demonstrated enhanced anti-tumor activity, characterized by increased IFN-γ and IL-17 expression and related responses. The role of IFN-γ is well-established in the induction of a protective effect against clinical and experimental HNSCC [40,41], and CD8^+^ T-cells are known to produce and become activated by this cytokine [42]. Further, we observed an elevation in the IFN-γ associated transcription factor *Stat1* in tumors of *Pik3cg^−/−^* mice. Previously, our group showed that STAT1 is essential to priming T-lymphocytes for anti-tumor immunity against aggressive HNSCC [43]. Our observation of higher expression of IFN-γ and STAT1 in *Pik3cg*^−/−^ mice at the tumor site indicates a potential anti-tumor cytotoxic response. We also observed increases in IL-17 expression by T-cells in the lymph nodes and spleen of tumor-bearing *Pik3cg*^−/−^ mice. Previous studies have reported similar increases in IL-17 production by *Pik3cg*^−/−^ mice in response to various stimuli [44]. While the role of IL-17 in HNSCC remains controversial, our group has previously shown that increased IL-17 is associated with lower metastatic burden and enhanced anti-tumor immunity in experimental murine HNSCC [34,45]. Further substantiating this observation was the elevated expression of *Cxcl9* and *Gzmb* in the primary tumors of *Pik3cg^−/−^* mice, which are respectively associated with chemotactic recruitment of lymphocytes and lymphocytic induction of tumor cytotoxicity [46,47]. Our findings are consistent with previous studies showing that p110γ blockade induces cytotoxic T-cell infiltration into and activity within the tumor microenvironment of solid tumors, including HNSCC and urothelial cancer [20,48]. Moreover, in the context of findings that IFN-γ and IL-17 are necessary for eliciting a therapeutic effect from PI3K inhibition in melanoma [32], our results suggest that host inhibition of PI3K p110γ enhances the anti-tumor activity of CD8^+^ T-cells via IFN-γ and/or IL-17 associated pathways. In summary, because the enhancement of CD8^+^ T-cell activity persisted even against non-immunogenic HNSCC, PI3K p110γ inhibition may serve as a host-directed approach to bolster the anti-tumor immune response.

It should be noted that our data indicate that PI3K p110γ inhibition was not sufficient to cause a substantial reduction in tumors in this highly aggressive murine HNSCC model. Therefore, PI3K p110γ inhibitors alone are unlikely to elicit a strongly therapeutic effect against highly aggressive clinical HNSCC. We further observed increased expression of PD-1 by T-cells in the tumor microenvironment of tumor-bearing *Pik3cg^−/−^* mice, which is associated with susceptibility to PD-L1-induced immune suppression [49]. We also observed a significant upregulation of PD-L1 among *Pik3cg^−/−^* G-MDSCs, M-MDSCs, and M2s at the primary tumor site and metastasized lymph nodes. These findings were surprising given our observation of increased cytotoxic markers at the tumor site. However, it is likely that this elevation in cytotoxicity counteracts the enhanced pro-tumor activity also present in *Pik3cg^−/−^* mice. A previous study showed that PI3Kγ inhibition using the small molecule inhibitor IPI-549 diminishes the accumulation of F4/80^+^CD206^+^ M2 populations in murine melanoma and breast cancer [50]. We did not observe an increase in M2 populations of *Pik3cg^−/−^* mice. However, we observed an increase in G-MDSC populations, which are known to significantly inhibit the anti-tumor activity of T-cells [51,52]. This finding supports previous observations on the role of the PI3K pathway in inhibiting MDSC immunosuppressive activity [53]. Taken together, our data show increases in PD-L1 expression and MDSC populations likely preclude the enhanced cytotoxic activity in *Pik3cg^−/−^* mice from making a significant impact on tumor growth. Given these findings, our results provide a rationale for targeting MDSC function or PD-L1 in conjunction with PI3K p110γ inhibition to promote cytotoxicity and enhanced efficacy of other therapeutics such as immune checkpoint inhibitors against poorly immunogenic HNSCC [54]. Future research will explore the efficacy of such combination therapies.

Recent studies investigating PI3K p110γ in the context of experimental murine HNSCC indicate that selective inhibitors show promise when administered in combination with PD-1 blockade. However, such studies were only performed in conjunction with HNSCC that had already been responsive to PD-1 inhibition alone [20]. About 60% of HNSCC patients are unresponsive to immune checkpoint therapy employing monoclonal antibodies against PD-1 or PD-L1 [37]. One reason for this low response rate is low tumor immunogenicity, marked by diminished infiltration of CD8^+^ T-cells and cytotoxic markers, such as IFN-γ and granzyme b, at the primary tumor site [55]. Our study shows that host inhibition of PI3K p110γ can potentially address this limitation, as tumor-bearing *Pik3cg^−/−^* mice demonstrated an increase in CD8^+^ T-cell infiltration into the tumor microenvironment of this poorly immunogenic HNSCC. Taken together, these data demonstrate that PI3K p110γ inhibition has the potential to enhance the anti-tumor response against HNSCC with low immunogenicity, especially in combination with checkpoint inhibitors.

The synergistic therapeutic effect of PD-1 and PI3K p110γ inhibition on HNSCC is currently being explored clinically [18]. However, the efficacy of PI3K p110γ inhibition with other immune checkpoint inhibitors has not been explored to a comparable extent. It has previously been shown that in vivo treatment of MOC2 tumors in a murine HNSCC model with PD-1 and CTLA-4 blockade does not confer increased anti-tumor immunity or increased presence of CD8^+^ tumor-infiltrating lymphocytes [28]. Interestingly, this poorly immunogenic murine HNSCC line has shown response to therapeutic inhibitors of mTOR but not PD-L1, though neither treatment increased infiltration of CD8^+^ TILs [56]. Our observation of increases in CD8^+^ T-cell presence and PD-L1 expression by immunosuppressive myeloid populations in the tumor microenvironment highlight counteracting forces that together fail to influence tumor development. Theoretically, disrupting this balance of cytotoxicity and immunosuppression could skew tumor development. Previously, dual p110δ/γ inhibition with the pharmaceutical IPI-145 has been shown to be ineffective in combination with anti-PD-L1 therapy in murine MOC2 HNSCC [26]. However, it has also been shown that dual inhibition of p110δ/γ abrogates CD8+ T-cell function while selective inhibition of p110δ or p110γ does not [19]. Therefore, because our findings indicate PI3K p110γ inhibition alone enhances CD8^+^ T-cell recruitment and preserves CD8^+^ T-cell function, this provides a rationale to combine PI3K p110γ inhibition with PD-L1 checkpoint inhibitors against poorly immunogenic HNSCC. Currently, combination therapy of anti-PD-L1 and IPI-549 is being explored as a treatment for patients with triple-negative breast cancer and renal cell carcinoma [57] and might prove effective in patients with poorly immunogenic HNSCC in the future.

## 4. Materials and Methods

### 4.1. Mouse Handling

Female *Pik3cg^−/−^* (*n* = 15) and *Pik3cg^+/+^* wild-type (WT; *n* = 15) C57BL/6 mice, age-matched at 7–8 weeks, were used for these studies. From each genotype group, mice were selected to receive cancer injections (n = 10) or act as healthy controls (*n* = 5). WT mice were acquired from Jackson Laboratories (Bar Harbor, ME, USA), and *Pik3cg^−/−^* mice were descendants of inbred mice acquired as described previously [58,59]. Animals were housed in Ohio State University animal facilities in accordance with state and federal guidelines provided by University Laboratory Animal Resources (ULAR). Animal experiments were approved by the Institutional Animal Care and Use Committee (Protocol #2018A00000054) and Institutional Biosafety Committee (IBC) of the Ohio State University.

### 4.2. Cancer Cell Line

MOC2 cell line, a highly aggressive mouse oral cancer cell line derived from the primary tumor in the floor-of-mouth of a *Cxcr3*^−/−^ mouse treated with the carcinogen DMBA, was obtained from Kerafast (Boston, MA, USA) and cultured according to recommended protocol at 37 °C and 5% CO_2_. Cells were grown to 75% confluence and passed after dissociation in TrypLE (Thermo Fisher Scientific; Waltham, MA, USA).

### 4.3. Antibodies

All antibodies used for flow cytometry and ELISA were purchased from BioLegend (San Diego, CA, USA). Primary monoclonal rat anti-mouse pan Cytokeratin (AE-1/AE-3) primary antibodies for IHC were acquired from BD Biosciences (San Jose, CA, USA), while horseradish peroxidase(HRP)-conjugated polyclonal goat anti-rat IgG secondary antibody was acquired from Southern Biotech (Birmingham, AL, USA). PIK3CG primary monoclonal antibody (OTI6H5) was purchased from Thermo Fisher Scientific.

### 4.4. Orthotopic HNSCC Model

We orthotopically injected 3 × 10^4^ MOC2 cells in 30 µL PBS into the right buccal mucosa of designated experimental C57BL/6 mice. Mice were weighed and tumor measurements were taken biweekly via calipers. Tumors were allowed to grow until meeting the threshold for termination. At terminal sacrifice, the lymph nodes, tumors, lungs, and spleens were harvested from experimental mice and processed for downstream analysis.

### 4.5. Flow Cytometry

Single cell suspensions were prepared from cervical lymph nodes, spleens, and tumors. Splenic cells were treated for 2 min with ACK Lysis Buffer to lyse erythrocytes. Tumor cells were digested with collagenase (Thermo Fisher Scientific; Waltham, MA) for 60 min at 37 °C before passing through nylon mesh filters. From each tissue, 10^6^ cells were processed with fluorochrome-conjugated antibodies for CD45, CD11b, Ly6G, Ly6C, F4/80, PD-L1, and CD206. Additionally, 2 × 10^6^ cells from each organ were treated with a cell activation cocktail containing PMA and ionomycin (Biolegend) for 6 h, after which they were stained with fluorophore-conjugated antibodies against CD3, CD4, CD8, and PD-1. Samples were subsequently intracellularly stained with fluorochrome-conjugated antibodies against IFN-γ and IL-17. Samples were run using a BD FACS Aria flow cytometer (BD Biosciences), and subsequent analysis was performed using FlowJo software (Tree Star, Inc., Ashland, OR, USA).

### 4.6. T-Cell Stimulation and ELISA

Splenic and lymph node cells from single cell suspensions were cultured on 96-well plates bound to αCD3 (1 µg/mL) and αCD28 (1 µg/mL) (BioLegend) for 72  h at 37 °C and 5% CO_2_. Following this incubation, supernatants were examined for IFN-γ, IL-2, IL-4, IL-6, IL-10, and IL-17 concentrations using cytokine ELISA. Capture and detection antibodies for these cytokines were purchased from Biolegend Streptavidin-alkaline phosphatase (BD Biosciences) and para-nitrophenyl phosphate (Thermo-Fisher Scientific) in glycine buffer were used for signal detection. Plates were read using a SpectraMax 190 plate reader (Molecular Devices; San Jose, CA, USA) at 405 nm. Concentrations of cytokines in supernatants were determined by extrapolation on the standard curve established by cytokine standard absorbance readings.

### 4.7. Quantitative Real-Time PCR

Portions of the lymph nodes, spleens, and tumors of experimental mice were placed in RNA-Later (Thermo Fisher Scientific) and stored at −80 C. Tissues were homogenized in 1 mL of TRIzol using a Bead Mill (VWR; Radnor, PA, USA). RNA was isolated using the Direct-zol RNA Miniprep kit (Zymo Research; Irvine, CA, USA). cDNA was prepared using 1μg RNA with High Capacity cDNA Reverse Transcription Kit (Applied Biosystems, Foster City, CA, USA). PCR amplification was performed in a CFX-384 Real-Time System (BioRad; Hercules, CA, USA). Samples were prepared in duplicate using PowerUp SYBR Green Master Mix (BioRad). Gene expression was normalized using β-actin (*Actb*) and glyceraldehyde-3-phosphate dehydrogenase (*Gapdh)* as reference genes. Fold inductions of *Ctla4, Cxcl9, Gzmb, Mhcii, Ifng, Il2, Il10, Il12, Il17, Pd1,* and *Pdl1* were calculated using the 2^-ΔΔct^ method. Primers were designed based on sequences previously verified by PrimerBank (https://pga.mgh.harvard.edu/primerbank/; accessed January 2020).

### 4.8. Histopathology and Immunohistochemistry

Primary tumors, draining cervical lymph nodes, and spleens were fixed in 10% neutral buffered formalin followed by paraffin embedding. Paraffin-embedded tissue sections were cut into 5 μm-thick cross-sections for histopathology and immunohistochemistry (IHC). Histopathological samples were stained with hematoxylin and eosin. For IHC, tissue sections were rehydrated in xylenes and graded ethanol before heat treatment to induce epitope retrieval. After antigen retrieval, IHC samples were blocked for 30 min in a 0.3% hydrogen peroxide/methanol to inhibit endogenous peroxidase activity. Tissue sections were blocked for 30 min in 10% normal goat serum prior to overnight incubation with monoclonal rat anti-mouse pan Cytokeratin (AE-1/AE-3) primary antibody. Samples were incubated for 1 h with HRP conjugated polyclonal goat anti-rat IgG secondary antibody. DAB peroxidase kit (Vector Laboratories Inc., Burlingame CA, USA) was used to detect the presence of HRP.

### 4.9. Statistics

Statistical analyses were performed using GraphPad Prism v8.0.2 (GraphPad Software, San Diego, CA, USA). Student’s *t*-test was used to determine statistically significant differences between groups with a *p*-value cut off of <0.05.

## 5. Conclusions

In conclusion, we demonstrate that host PI3K p110γ inhibition does not affect the growth or metastasis of aggressive murine HNSCC with poor immunogenicity, though it has potent immunomodulatory effects that lead to elevations in both cytotoxic and immunosuppressive responses. These altered responses are marked by enhanced infiltration and activity of both anti-tumor CD8^+^ T-lymphocytes and immunosuppressive MDSCs in the tumor microenvironment. These results might explain why PI3K inhibition response rates are suboptimal when used as a monotherapy against patients with solid tumors. Nevertheless, our findings show that PI3K p110γ inhibition may serve as a strategy for potentially enhancing the cytotoxic response against poorly immunogenic HNSCC and likely potentiates HNSCC treatment when used in combination with selective checkpoint inhibitors. Future research will explore the clinical potential of PI3K p110γ inhibition in combination with therapies that inhibit host immunosuppressive pathways.

## Data Availability

Data is contained within the article. The data presented in this study are available in this article.
